# Thinking twice: examining gender differences in repetitive negative thinking across the adult lifespan

**DOI:** 10.3389/fpsyg.2023.1239112

**Published:** 2023-11-08

**Authors:** Kieren J. Lilly, Chloe Howard, Elena Zubielevitch, Chris G. Sibley

**Affiliations:** ^1^School of Psychology, University of Auckland, Auckland, New Zealand; ^2^Business School, The University of Queensland, Brisbane, QLD, Australia

**Keywords:** cohort-sequential design, rumination, gender differences, longitudinal analysis, generational differences

## Abstract

**Background:**

A wealth of literature shows that women report greater levels of repetitive negative thinking, particularly rumination, than men in adolescence and adulthood. However, little research has examined how these gender differences develop or change across the entire adult lifespan.

**Methods:**

The present study addresses these oversights using a nationwide longitudinal probability sample of adults over 12 annual assessment points (*N* = 64,901; *Mage* = 42.50, range 18–81; 62.9% women) and a single-item measure of global repetitive negative thinking. Critically, we use multigroup cohort-sequential latent growth modeling to determine whether changes in this construct over time are due to (a) normative aging, (b) generational differences associated with the historical period one was born and raised in, or (c) a combination of these processes.

**Results:**

Our results reveal that rumination peaks in young adulthood for both women and men but declines steadily thereafter, reaching its lowest levels at the end of the adult lifespan. That said, some gender and cohort differences emerged, with young women—particularly young cohorts—reporting higher levels of rumination than their male counterparts and older birth cohorts.

**Discussion:**

Our study suggests that gender differences in rumination may be most prevalent among young birth cohorts, though future research is needed to elucidate the mechanisms underlying these processes.

## 1. Introduction

Rumination refers to repetitive, persistent, and passive thinking about the symptoms, circumstances, causes, and consequences of depressed mood or distress (Nolen-Hoeksema, 1991; Watkins and Roberts, 2020). Rumination has been implicated in the onset of several mental health conditions, including depression (see Nolen-Hoeksema and Morrow, 1993; Lyubomirsky et al., 2015), anxiety (Olatunji et al., 2013; Parthi and Kaur, 2022), and eating disorders (for a recent meta-analytic review, see Rickerby et al., 2022). Critically, a robust body of literature posits rumination as a key factor contributing to the known gender differences in these disorders (e.g., Nolen-Hoeksema, 1994; Hyde et al., 2008; Johnson and Whisman, 2013; Espinosa et al., 2022). For example, women consistently report higher rates of depression than men (World Health Organisation, 2017), and research suggests that this pattern may occur because of emerging gender differences in rumination during early adolescence (Nolen-Hoeksema, 1994; Jose and Brown, 2008).

Yet, although gender differences in rumination are firmly established, most research focuses on adolescent and young adult samples (e.g., Nolen-Hoeksema, 1994; Hampel and Petermann, 2005; Espinosa et al., 2022). Thus, it remains unknown whether the gender disparity in rumination continues into later adulthood. In the current study, we remedy this gap by examining the development of rumination across the adult lifespan using 12 annual waves of a nationwide random panel sample. To do so, we model responses to a single-item measure developed to assess general levels of negative repetitive thinking, in line with the conceptual definition of rumination developed by Nolen-Hoeksema et al. (1993). It is important to note there are different forms of repetitive negative thinking that, while similar in terms of process and function, differ in temporal orientation (i.e., repetitive negative thinking about the past versus the future, see Ehring and Watkins, 2008; Nolen-Hoeksema et al., 2008). The present study, however, assesses the global repetitive negative thinking that underlies rumination as directly as possible without specifying the temporal orientation. While we acknowledge the complexities of different forms of repetitive negative thinking, the present study refers to rumination as the focal framework for understanding gender differences in repetitive negative thinking across the lifespan.

Critically, we utilize cohort-sequential latent growth curve modeling (Prinzie and Onghena, 2005) to investigate how rumination develops and changes across adulthood and compare whether this trajectory differs between women and men. Moreover, we also examine the possibility that gender differences in rumination are affected by historical processes (i.e., generational or cohort differences). By examining these processes, we can elucidate the gender differences (or lack thereof) in general levels of rumination across adulthood, as well as the age groups and cohorts most affected by these differences. We begin by reviewing prior research examining gender, age, and cohort differences in response styles, particularly in rumination, before outlining the current study and our hypotheses.

Nolen-Hoeksema (1994) provides a model for understanding gender differences in depression whereby one’s response style—either passive or active—affects their depressed mood (see also Nolen-Hoeksema, 1991). A passive style of responding to depression is characterized by rumination, which likely worsens or maintains depressed mood by hindering one’s ability to generate solutions to interpersonal problems (e.g., Lyubomirsky and Nolen-Hoeksema, 1995) and increasing the salience of negative emotions and thoughts (e.g., Teasdale, 1983). In contrast, an active response style typically involves efforts to problem-solve and change or remove the stressor (Tamres et al., 2002), which may reduce levels of depression and anxiety following stressful events (Nolen-Hoeksema, 1991). Importantly, women are more likely than men to engage in ruminative responses (Johnson and Whisman, 2013) which, in turn, may partially explain gender differences in depression (Nolen-Hoeksema and Aldao, 2011).

Gender differences in rumination likely emerge in pre-adolescence but become more pronounced due to the challenges and stressors of adolescence (see also Nolen-Hoeksema, 1994). Cross-sectional research tentatively corroborates this hypothesis, showing that gender differences in rumination appear around age 12 (Hampel and Petermann, 2005; Jose and Brown, 2008) and remain into adulthood (see Johnson and Whisman, 2013). Greater exposure to uncontrollable, gendered life events may explain why women and young girls are more at risk of rumination (Nolen-Hoeksema et al., 1999; Michl et al., 2013). For example, adolescent girls report more interpersonal stressors than adolescent boys (e.g., Hankin et al., 2007) and heightened reactivity to these stressors (e.g., Shih et al., 2006). In adulthood, women also report higher rates of life events than men (Kessler and McLeod, 1984; Norris, 1992; Kendler et al., 2001; Turner and Avison, 2003; Howard et al., 2022), which is likely to be a risk factor for greater rumination.

That said, research has primarily focused on establishing when gender differences *emerge* (see Nolen-Hoeksema, 1994; Nolen-Hoeksema and Girgus, 1994; Hyde et al., 2008; Jose and Brown, 2008), meaning that little is known about whether these differences continue over the lifespan. Nevertheless, some cross-sectional research suggests that women report higher levels of rumination and negative self-focus across early, mid, and later adult age groups (e.g., Leach et al., 2008; Nolen-Hoeksema and Aldao, 2011; Meléndez et al., 2012; Zimmermann and Iwanski, 2014; Chen et al., 2020). Providing indirect evidence, studies also show that women consistently report higher rates of depression, anxiety, and other conditions associated with ruminative strategies (e.g., Mirowsky and Schieman, 2008; McLean and Anderson, 2009; Salk et al., 2017; Twenge et al., 2019). Although these gender differences are typically smaller than in adolescence and early adulthood (Johnson and Whisman, 2013; Olatunji et al., 2013), these studies suggest that gender differences in rumination may be relatively persistent across the lifespan.

Yet, some research suggests that gender differences in rumination may *reduce* in magnitude rather than persist as people grow older. Indeed, some studies have found smaller or negligible gender differences in depressive disorders among older adults (e.g., Bebbington et al., 2003; Pachana et al., 2012). Thus, rumination may follow a similar trajectory, whereby gender differences become less marked across the adult lifespan. However, no research to our knowledge has explicitly examined how rumination changes and develops across the lifespan. Such longitudinal investigations are needed to determine whether women consistently report higher levels of rumination than men or whether these differences are localized at particular life stages. In doing so, we can determine the specific gender and age groups high in rumination and better prioritize resources to minimize the mental health consequences of rumination.

Although the present study primarily examines lifespan trajectories of gender differences in rumination, it is difficult to disentangle lifespan explanations from the effects of sociohistorical change (i.e., effects due to shifts in culture over time and social differences between generations). After all, the interactions between biological, cognitive, and affective vulnerabilities and adverse life events produce gender differences in mental health (see Hyde et al., 2008; Leach et al., 2008; Hamilton et al., 2015). As such, one’s social and interpersonal context is integral to understanding differences in rumination, and different *generations* may demonstrate (a) larger (or smaller) gender differences in rumination and (b) distinct *trajectories* of rumination over time. Accordingly, longitudinal modeling of people across different generations is needed to identify and disentangle cohort differences (or lack thereof) from normative age-related changes in rumination over time.

As mentioned, adolescence is a critical period in the lifespan for rumination due to the uniquely associated physiological changes, life circumstances, and stressors (Nolen-Hoeksema, 1994). Yet, emerging and young adults also experience a myriad of unique stressors that may also be relevant to understanding rumination in early adulthood (Schulenberg et al., 2004). Emerging adults (age 18–25; see Arnett, 2000; Arnett, 2007) experience a period of profound change characterized by a transition to greater independence and exploration. Likewise, young adults enter adulthood expecting to complete several personal goals, such as completing education or entering a long-term relationship. Regarding age-specific events, young adults report more stressors than older adults, particularly in relation to education, interpersonal relationships and sexuality, and traumatic events (e.g., assault and suicide; see Norris, 1992; Hatch and Dohrenwend, 2007; Howard et al., 2022).

Conversely, research suggests that older adults (a) endorse more positive forms of emotional regulation and (b) have a more comprehensive range of emotional regulation strategies than young adults (e.g., Blanchard-Fields et al., 1997, 2007; Charles, 2010). For example, older adults experience unique stressors compared to young adults that often center around health, bereavement, and family (Murrel et al., 1984) and, as they age, become more practiced at coping with these stressors (Blanchard-Fields et al., 1997, 2007). In contrast, young adults report greater levels of rumination than older adults (Thomsen et al., 2005; Sütterlin et al., 2012; Zimmermann and Iwanski, 2014), and incidences of mood disorders peak in early adulthood and decline thereafter (e.g., Twenge et al., 2019). Thus, in the present study, we should see a normative trajectory of rumination across the lifespan whereby the highest levels of rumination occur in emerging-to-young adulthood and subsequently decline throughout middle-to-late adulthood.

While rumination should generally follow a normative developmental process, we do expect some cohort-based differences over time. Indeed, historical events and societal changes unfolding during one’s formative years are incredibly important for forming generation-specific attitudes and cognitions (Campbell et al., 2015). For example, children growing up in the 1940s post-World War II period experienced a fundamentally different sociopolitical culture than children in the 1990s. In particular, attitudes toward mental health have changed markedly (e.g., Mojtabai, 2007; Angermeyer et al., 2017), with shifts toward anti-stigma and ‘awareness’ campaigns and media coverage (e.g., Rhydderch et al., 2016). Accordingly, research suggests that young birth cohorts endorse more progressive attitudes toward mental health than older cohorts (e.g., Pescosolido et al., 2021) and greater support for treatment and services (e.g., Pescosolido et al., 2010).

Moreover, older adults were socialized in a time when passive responding styles (i.e., rumination) were discouraged, whereas young generations were encouraged and socialized to recognize their feelings and self-explore (Twenge and Campbell, 2009; Nolen-Hoeksema and Aldao, 2011). This suggests that younger generations may be more inclined to report negative thoughts, feelings, and behaviors than their predecessors. Thus, while young adults may report higher levels of rumination (relative to older adults) due to a normative developmental difference, the youngest adult *cohorts* may report the highest levels of rumination due to the shifting attitudes toward mental health and, thus, shifting expectations for young adults. Given the assertion that young women, on average, report higher levels of rumination than men (see Johnson and Whisman, 2013) and experience more gender-specific life events (e.g., Norris, 1992; Turner and Avison, 2003), we expect young cohorts of *women*, in particular, to report high levels of rumination.

While research has established consistent gender differences in rumination (Nolen-Hoeksema, 1987; Nolen-Hoeksema and Girgus, 1994; Nolen-Hoeksema and Aldao, 2011), little is known of how these differences persist across the lifespan. Moreover, while research suggests younger (relative to older) adults report greater levels of rumination, research has yet to examine the potential developmental *and* generational differences in rumination over time. Given the known associations rumination has with myriad mental health conditions (e.g., Olatunji et al., 2013; Rickerby et al., 2022), the potential aging and cohort trajectories of rumination across time warrant exploration.

The current study examines the development of rumination across the adult lifespan (from age 18 to 81) and whether this trajectory differs among women and men using 12 annual waves of a large, nationwide random probability sample of adults. Specifically, we use a single-item measure of rumination designed to assess general levels of negative repetitive thinking across the lifespan. While there are several forms of repetitive negative thinking (see Ehring and Watkins, 2008), including different forms of *rumination* (e.g., brooding, reflective, intrusive, and deliberate rumination; see García et al., 2017) across different contexts (e.g., interpersonal or body image domains; Mezulis et al., 2002), our measure mirrors the conceptual definition developed by Nolen-Hoeksema et al. (1993). By using this measure, we aim to identify general ruminative styles of responding in the general population, rather than domain-specific rumination.

Critically, we use cohort-sequential latent growth curve modeling, which allows us to assess age versus cohort-related change in rumination over time (for recent examples, see Milfont et al., 2021; Zubielevitch et al., 2023). More specifically, we estimate three separate cohort-sequential latent growth models that reflect three possible processes underlying the development of rumination across the lifespan: (a) an aging model that assumes common developmental trends across the lifespan, (b) a period model that assumes common trends across birth cohorts due to shared societal change, and (c) a cohort model that assumes differences between birth cohorts due to the unique contexts associated with their formative years. While cohort-sequential models by no means entirely distinguish aging and cohort effects, our ability to model multiple birth cohorts over 12 years is a close approximation. Perhaps more importantly, we estimate models for both women and men, which allows us to inspect gender differences in these trends across the lifespan.

Given the mixed consensus on the persistence of gender differences in rumination across the lifespan, we make no specific predictions for the magnitude of differences between women and men across adulthood. We do, however, expect differences to be *most* pronounced between young women and men, given the emergence of differences in rumination in adolescence (Nolen-Hoeksema and Girgus, 1994; Jose and Brown, 2008) and continuing differences in young adulthood (Thomsen et al., 2005; Leach et al., 2008). Moreover, the extant literature suggests normative age-based differences in rumination and regulation strategies for both women and men (e.g., Blanchard-Fields et al., 2007; Sütterlin et al., 2012; Twenge et al., 2019). We thus expect rumination to generally follow a normative aging process—specifically, the highest levels of rumination should occur in emerging-to-young adulthood and subsequently decline throughout middle-to-late adulthood. That said, given the shifting attitudes toward mental health among young cohorts (Mojtabai, 2007; Angermeyer et al., 2017), we also expect young adult cohorts—particularly young cohorts of women—to report the highest levels of rumination across time. Overall, we aim to elucidate when and how rumination differs across the lifespan, as well as the age groups and cohorts most susceptible to ruminative thinking.

## 2. Methods

### 2.1. Participants and sampling procedure

We use data from the New Zealand Attitudes and Values Study (NZAVS)—an ongoing, nationwide longitudinal panel study that began in 2009. Although the NZAVS began in 2009, we first assessed rumination at Time 2 (2010). As such, we focus on participants who responded to one or more assessment occasions from Time 2 (2010) to Time 13 (2021)—the most recently completed wave of the NZAVS (*N_total_* = 64,901). Notably, the NZAVS sample has relatively low rates of attrition, with 33.52% of participants at Time 13 (2021) retained from Time 1 (2009) and good wave-to-wave retention (67.9–85.6%; see Sibley, 2023). Most of our participants were women (62.9%), and the average age of participants at Time 2 was 42.50 years (*SD* = 13.16; range_total_: 18–81 years). Concerning ethnicity, most of the sample were New Zealand European (77.8%) or Māori (12.7%), with the remaining participants identifying as Asian (5.1%) and Pasifika (2.7%). Our sample also represents the New Zealand population in socioeconomic status as measured using the New Zealand Deprivation Index (*M* = 4.89, *SD* = 2.79, range: 1–10), a decile-based measure assessing neighborhood deprivation across the country from 1 (most affluent) to 10 (most deprived; see Atkinson et al., 2019). Sibley (2023) provides full details of the sampling procedure, demographic information, methodology, and ethics approvals for the NZAVS (see also the OSF page: https://osf.io/75snb/).

We grouped participants into 5-year cohorts for our analyses based on their birth year and gender. Table 1 displays each birth cohort’s sample size and age by gender across the 12 assessment occasions.

**Table 1:** Age and sample size for birth cohorts by gender.

			Sample sizes	
Birth cohorts	Age at Time 2 (~2010)	Age at Time 13 (~2021)	Women *n*	Men *n*
1995–1991	18	26	2,615	1,223
1990–1986	19	31	3,153	1,529
1985–1981	24	36	3,283	1,585
1980–1976	29	41	3,713	1,878
1975–1971	34	46	4,507	2,332
1970–1966	39	51	5,063	2,859
1965–1961	44	56	5,821	3,530
1960–1956	49	61	6,039	4,093
1955–1951	54	66	4,245	3,231
1950–1946	59	71	1,337	983
1945–1941	64	76	708	577
1940–1936	69	81	309	288
			40,793	24,108
*N_total_*			64,901	

Youngest age in birth cohort taken as an indication of participants’ age at Time 2.

### 2.2. Measures

#### 2.2.1. Rumination

Rumination was measured at each wave using a single item: “During the last 30 days, how often did…you have negative thoughts that repeated over and over?” (Matika et al., 2017). This item was developed for the NZAVS based on the conceptual definition of rumination described by Nolen-Hoeksema et al. (1993). The item was measured on a scale from 0 (*None of the time*) to 4 (*All of the time*), with higher scores indicating higher levels of rumination. Table 2 displays the bivariate correlations between our measures of rumination at each time point.

**Table 2:** Descriptive statistics and bivariate correlations between our focal variables.

	1.	2.	3.	4.	5.	6.	7.	8.	9.	10.	11.	12.	13.	14.
Rumination_T2_	–													
Rumination_T3_	0.554^***^	–												
Rumination_T4_	0.532^***^	0.528^***^	–											
Rumination_T5_	0.487^***^	0.507^***^	0.559^***^	–										
Rumination_T6_	0.491^***^	0.505^***^	0.524^***^	0.562^***^	–									
Rumination_T7_	0.473^***^	0.482^***^	0.501^***^	0.540^***^	0.587^***^	–								
Rumination_T8_	0.435^***^	0.475^***^	0.498^***^	0.515^***^	0.557^***^	0.580^***^	–							
Rumination_T9_	0.481^***^	0.488^***^	0.491^***^	0.509^***^	0.533^***^	0.546^***^	0.586^***^	–						
Rumination_T10_	0.445^***^	0.483^***^	0.474^***^	0.475^***^	0.512^***^	0.517^***^	0.559^***^	0.586^***^	–					
Rumination_T11_	0.445^***^	0.468^***^	0.462^***^	0.465^***^	0.497^***^	0.501^***^	0.534^***^	0.567^***^	0.578^***^	–				
Rumination_T12_	0.443^***^	0.480^***^	0.471^***^	0.474^***^	0.490^***^	0.504^***^	0.521^***^	0.536^***^	0.542^***^	0.585^***^	–			
Rumination_T13_	0.435^***^	0.430^***^	0.442^***^	0.455^***^	0.494^***^	0.496^***^	0.514^***^	0.518^***^	0.524^***^	0.553^***^	0.589^***^	–		
Gender^a^	−0.002	−0.015	−0.008	0.001	−0.015	−0.011	−0.007	−0.013	−0.009	−0.015^**^	−0.017^**^	−0.032^***^	–	
Age^b^	−0.165^***^	−0.229^***^	−0.200^***^	−0.187^***^	−0.183^***^	−0.165^***^	−0.191^***^	−0.192^***^	−0.192^***^	−0.192^***^	−0.192^***^	−0.202^***^	0.081^***^	–
*M*	0.78	0.84	0.79	0.79	0.75	0.75	0.80	0.76	0.82	0.81	0.79	0.80	0.37	42.50
*SD*	0.97	1.00	0.98	0.98	0.95	0.94	0.98	0.94	0.99	0.97	0.96	0.96	0.48	13.16
Sample size	4,119	6,424	11,663	17,501	15,428	13,612	21,172	16,564	45,249	40,636	36,804	32,346	64,901	64,901

^a^Dummy-coded (0 = woman, 1 = man). ^b^Age at Time 2 (2010). ^*^p < 0.05, ^**^p < 0.01, ^***^p < 0.001.

#### 2.2.2. Gender

Gender was measured differently over time following a move to a more inclusive measure. In Times 2–5, gender was measured by asking participants a forced binary choice question (“Are you male or female?). From Time 6 onwards, gender was measured using an open-ended question, “What is your gender?” (Fraser et al., 2020). For our analyses, we dummy-coded participants’ gender (0 = woman, 1 = man) and excluded gender-diverse participants due to the small sample size (0.5% of the total NZAVS sample).

### 2.3. Analytic approach

To examine the trajectory of rumination over time—and whether this trajectory differs between women and men—we estimated multigroup cohort-sequential latent growth models based on 5-year birth cohorts using *Mplus* version 8.8 (Muthén and Muthén, 1998-2023). While traditional latent growth models permit us to examine mean growth trajectories for a particular cohort, cohort-sequential designs estimate mean growth trajectories for different cohorts *simultaneously*, allowing us to identify (a) common developmental trends across adulthood (i.e., overlapping estimates in different birth cohorts), and (b) any potential differences between birth cohorts over time (see Prinzie and Onghena, 2005).

While this approach has been well-documented elsewhere (for a recent example, see Zubielevitch et al., 2023), we provide a brief overview here. First, we sorted our sample into 12 sequential birth cohorts based on birth year, spanning 1995 to 1936 (ages 18 to 81). We used the youngest age within each birth cohort to indicate participants’ age at Time 2 (2010); for example, the 1990–1986 cohort reflected change from ages 19 to 31. There is one exception to this—the 1995–1991 birth cohort spanned a shorter time period (from age 18 to 26) as the youngest theoretical age (15) is beyond the observed range of ages in the NZAVS, which only surveyed those over the age of 18. Additionally, we separated each birth cohort by gender to model change for both women and men. To handle missing data, we conducted all analyses using full information maximum likelihood estimation (FIML; see Enders and Bandalos, 2001).

We first estimated an aging model that examined trajectories of rumination across the lifespan as a function of normative developmental change. That is, the model assumes that different birth cohorts will have comparable *initial* levels of rumination and rates of change across time. Specifically, the model constrains the intercepts and slopes to equality across all 12 birth cohorts. To account for possible curvilinear rates of change over time, we estimated linear and quadratic slopes in our analyses. The model was then age-centered at 45 years and conditioned by age so that we could plot the point estimates across ages 18 to 81 and identify any age-related trends in rumination across the adult lifespan for both women and men.

We then estimated a period model—an intermediate model that allows birth cohorts to differ in their initial levels of rumination (i.e., the intercepts) but constrains the rate of change to equality across birth cohorts. Accordingly, we constrained the slopes to equality but freely estimated intercepts for each birth cohort. Finally, we estimated a cohort model that examined the possibility that generational differences uniquely affect rumination across the lifespan. That is, initial levels of rumination *and* rates of change over time differ across birth cohorts due to the contextual factors associated with the period in which they were born. As such, we freed the intercepts and the slopes for each birth cohort. As with our aging model, we conditioned the point estimates by age to plot the trends for each birth cohort across the12 annual assessments.

To assess model fit and determine whether changes in rumination across the adulthood lifespan reflect aging or cohort effects, we inspected widely used model fit indices for each model. However, determining aging versus cohort processes requires more nuance than provided by global fit indices (see Steiger, 2007). Given this, we use global fit indices *and* our plots of the aging and cohort estimates to holistically inspect whether birth cohort estimates broadly follow an aging trend or whether there are unique trajectories across birth cohorts over time.

## 3. Results

### 3.1. Aging model

**Figure 1:**
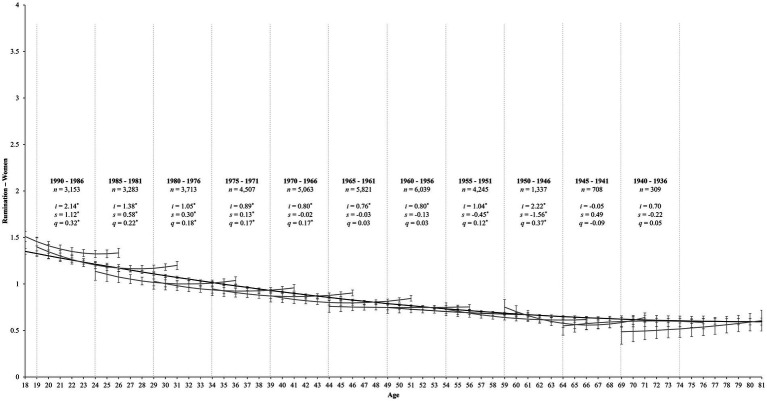
Change trajectories and comparisons of aging and cohort models for rumination among women. The aging trajectory for rumination among women is shown by the black line from ages 18 to 81. The gray lines within each 5-year birth cohort panel demonstrate longitudinal change in rumination over 12 years for each birth cohort, estimating intercepts (i), linear slopes (s), and quadratic slopes (q). Error bars reflect 95% confidence intervals for each point estimate. Due to graphical constraints, we plot the estimates for the 1995–1991 birth cohort (ages 18–26, *n* = 2,615) but could not display them: *i* = 3.27^*^, *s* = 1.89^*^, *q* = 0.46^*^. ^*^p < 0.05.

**Figure 2:**
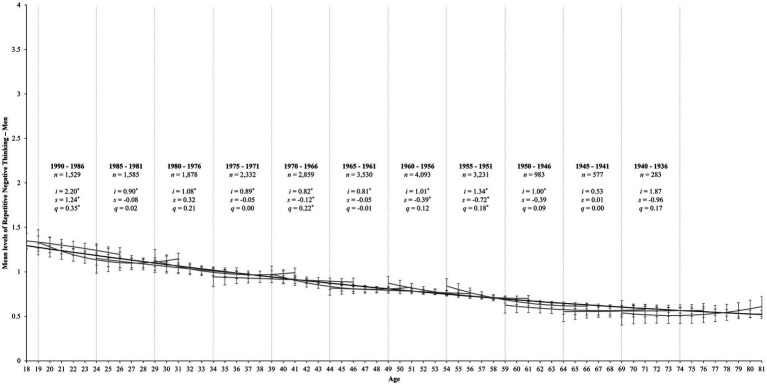
Change trajectories and comparisons of aging and cohort models for rumination among men. The aging trajectory for rumination among men is shown by the black line from ages 18 to 81. The gray lines within each 5-year birth cohort panel demonstrate longitudinal change in rumination over 12 years for each birth cohort, estimating intercepts (i), linear slopes (s), and quadratic slopes (q). Error bars reflect 95% confidence intervals for each point estimate. Due to graphical constraints, we plot the estimates for the 1995–1991 birth cohort (ages 18–26, *n* = 1,223) but could not display them: *i* = 0.51, *s* = −0.48, *q* = −0.06. ^*^p < 0.05.

**Figure 3:**
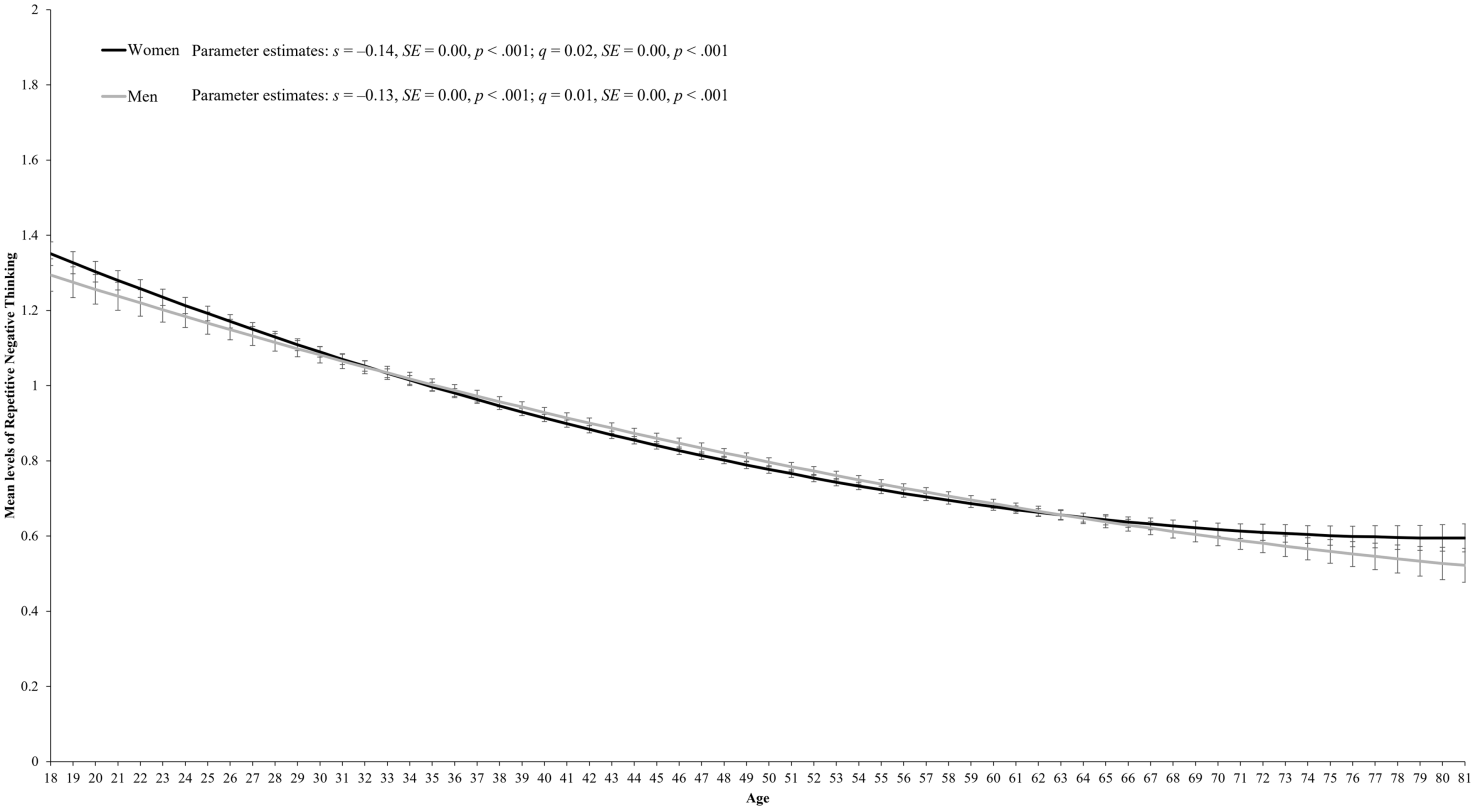
Comparisons of the aging trajectories for rumination among women (*N* = 40,793) and men (*N* = 24.108). The black line depicts the aging trajectory for women, while the gray line depicts the aging trajectory for men. The graph depicts a 0–2 scale to facilitate interpretation of gender differences, but rumination was measured on a 0–4 scale. Error bars reflect 95% confidence intervals for each point estimate.

Table 3 shows the aging model fits these data well [χ^2^_(2150)_ = 9930.67, *p* < 0.001, CFI = 0.92, RMSEA = 0.04, SRMR = 0.07]. The parameter estimates that best fit all birth cohorts are displayed in Table 4. For women, Table 4 reveals a significant curvilinear change in rumination over time (*s* = −0.14, *SE* = 0.00, *p* < 0.001; *q* = 0.02, *SE* = 0.00, *p* < 0.001). Rumination among men displayed a similar aging trend (*s* = −0.13, *SE* = 0.00, *p* < 0.001; *q* = 0.01, *SE* = 0.00, *p* < 0.001). Indeed, an inspection of the aging lines in Figures 1, 2 (i.e., the black lines) reveals a decline in rumination from age 18 onwards but that this rate of change slowed over time for both women and men. However, a follow-up Wald test did reveal a small but significant difference between the trajectories of women and men across time [*Wald*_(3)_ = 9.73, *p* = 0.021]. These subtle gender differences are displayed in Figure 3 and reveal greater curvilinear changes in rumination among women than men.

**Table 3:** Model fit for aging, period, and cohort models.

Model	χ^2^	d*f*	*p*	CFI	RMSEA	SRMR	AIC	BIC	aBIC
Aging	9930.67	2,150	<0.001	0.919	0.037	0.069	623827.89	623918.69	623886.91
Period	9616.47	2,128	<0.001	0.922	0.036	0.068	623557.68	623848.26	623746.57
Cohort	9422.57	2,084	<0.001	0.924	0.041	0.067	623451.79	624141.91	623900.38

Model comparison	Δχ^2^	Δd*f*	*p*	ΔCFI	ΔRMSEA	ΔSRMR	ΔAIC	ΔBIC	ΔaBIC
Age–period	314.20	22	<0.001	0.003	0.001	0.001	270.20	70.43	140.35
Period–cohort	193.90	44	<0.001	0.002	−0.005	0.001	105.90	−293.65	−153.82

χ^2^, chi-square; df, degrees of freedom; *p*, *p*-value; CFI, comparative fit index; RMSEA, root mean square error of approximation; SRMR, standardized root mean square residual.

**Table 4:** Parameter coefficients for the aging models for rumination by gender.

	Est.	*SE*	Est./SE	*p*	95% CI		
					LB	UB	Variances
Women							
Intercept (*i*)	0.84	0.01	166.79	<0.001	0.83	0.85	0.51^***^
Linear Slope (*s*)	−0.14	0.00	−45.17	<0.001	−0.14	−0.13	0.02^***^
Quadratic slope (*q*)	0.02	0.00	11.02	<0.001	0.02	0.02	0.00
Men							
Intercept (*i*)	0.86	0.01	129.72	<0.001	0.85	0.87	0.51^***^
Linear Slope (*s*)	−0.13	0.00	−31.42	<0.001	−0.14	−0.12	0.02^***^
Quadratic slope (*q*)	0.01	0.00	4.74	<0.001	0.01	0.02	0.00

Variances constrained to equality across birth cohorts and genders. CI, confidence interval; LB, lower bound; UB, upper bound. ^***^p < 0.001.

### 3.2. Period model

The period model also fits these data well [χ^2^_(2128)_ = 9616.47, *p* < 0.001, CFI = 0.92, RMSEA = 0.04, SRMR = 0.07; Table 3]. Table 5 displays the parameter estimates for this model and reveals that the values for the freely estimated intercepts were generally lower for each successive birth cohort for both women and men. That is, young birth cohorts had higher mean levels of rumination than older birth cohorts, irrespective of gender. Interestingly, only women in the two youngest birth cohorts (1995–1986) had higher initial mean levels of rumination than men in the corresponding birth cohorts (i.e., the confidence intervals of the estimates did not overlap). Similar to the aging model, the period model suggests a linear decline in rumination among women over time (*s* = −0.03, *SE* = 0.01, *p* < 0.001) but that this rate of change slowed over time (*q* = 0.01, *SE* = 0.00, *p* < 0.001). A similar trend emerged for men over time, albeit with greater linear declines in rumination across the lifespan (*s* = −0.08, *SE* = 0.01, *p* < 0.001; *q* = 0.01, *SE* = 0.00, *p* = 0.015).

**Table 5:** Parameter estimates for the period model for rumination by gender.

		Women						Men					
Birth cohort		Est.	*SE*	Est./SE	*p*	95% CI		Est.	*SE*	Est./SE	*p*	95% CI	
						LB	UB					LB	UB
1995–1991		**1.24** ^***^	0.03	40.76	<0.001	1.18	1.30	**1.04** ^***^	0.05	22.81	<0.001	0.95	1.13
1990–1986	Intercepts	**1.10** ^***^	0.02	46.33	<0.001	1.06	1.15	**0.96** ^***^	0.04	27.24	<0.001	0.89	1.03
1985–1981	freely	0.96^***^	0.02	50.55	<0.001	0.93	1.00	0.91^***^	0.03	32.61	<0.001	0.86	0.97
1980–1976	estimated	0.91^***^	0.02	59.55	<0.001	0.88	0.94	0.92^***^	0.02	41.73	<0.001	0.87	0.96
1975–1971		0.87^***^	0.01	70.30	<0.001	0.85	0.90	0.88^***^	0.02	50.57	<0.001	0.85	0.92
1970–1966		0.83^***^	0.01	72.51	<0.001	0.80	0.85	0.84^***^	0.02	55.23	<0.001	0.81	0.87
1965–1961		0.77^***^	0.01	66.45	<0.001	0.75	0.79	0.83^***^	0.02	55.15	<0.001	0.80	0.86
1960–1956		0.71^***^	0.01	52.38	<0.001	0.68	0.73	0.81^***^	0.02	47.96	<0.001	0.78	0.84
1955–1951		0.65^***^	0.02	36.30	<0.001	0.61	0.68	0.75^***^	0.02	35.07	<0.001	0.71	0.80
1950–1946		0.63^***^	0.03	23.59	<0.001	0.58	0.68	0.71^***^	0.03	22.71	<0.001	0.65	0.77
1945–1941		0.61^***^	0.04	17.10	<0.001	0.54	0.68	0.71^***^	0.04	17.68	<0.001	0.63	0.79
1940–1936		0.55^***^	0.05	10.97	<0.001	0.45	0.65	0.70^***^	0.05	13.08	<0.001	0.60	0.81
All cohorts	Linear slope constrained	−0.03^***^	0.01	−4.33	<0.001	−0.05	−0.02	−0.08^***^	0.01	−7.65	<0.001	−0.10	−0.06
All cohorts	Quadratic slope constrained	0.01^***^	0.00	3.49	<0.001	0.00	0.01	0.01^*^	0.00	2.42	0.015	0.00	0.02

Significant gender differences within birth cohorts denoted in bold. Variances constrained to equality across birth cohorts and genders (*i* = 0.51^***^, SE = 0.01; *s* = 0.02^***^, *SE* = 0.00; *q* = 0.00, *SE* = 0.00). ^***^p < 0.001, ^**^p < 0.01, ^*^p < 0.05.

### 3.3. Cohort model

Finally, the cohort model also fits these data well [χ^2^_(2084)_ = 9930.67, *p* < 0.001, CFI = 0.92, RMSEA = 0.04, SRMR = 0.07; Table 3]. In terms of women, Table 6 reveals that 8 out of 12 birth cohorts showed significant changes in rumination over 12 years (*p*s < 0.05). The gray lines in Figure 1 display the trends for each birth cohort and reveal that the 1995–1991 (*s* = 1.89, *SE* = 0.51, *p* < 0.001; *q* = 0.46, *SE* = 0.11, *p* < 0.001), 1990–1986 (*s* = 1.12, *SE* = 0.31, *p* < 0.001; *q* = 0.32, *SE* = 0.08, *p* < 0.001), 1985–1981 (*s* = 0.58, *SE* = 0.22, *p* = 0.008; *q* = 0.22, *SE* = 0.08, *p* = 0.006), 1980–1976 (*s* = 0.30, *SE* = 0.12, *p* = 0.016; *q* = 0.18, *SE* = 0.07, *p* = 0.012), and 1975–1971 (*s* = 0.13, *SE* = 0.05, *p* = 0.010; *q* = 0.17, *SE* = 0.06, *p* = 0.009) birth cohorts experienced curvilinear increases in rumination over time. The 1970–1966 birth cohort showed positive curvilinear change (*q* = 0.17, *SE* = 0.06, *p* = 0.003). Finally, the 1955–1951 (*s* = −0.45, *SE* = 0.20, *p* = 0.001; *q* = 0.20, *SE* = 0.06, *p* = 0.044) and 1950–1946 (*s* = −1.56, *SE* = 0.35, *p* < 0.001; *q* = 0.37, *SE* = 0.08, *p* < 0.001) birth cohorts showed linear *decreases* in rumination over time but that this rate of change slowed over time. The remaining cohorts showed no significant change in rumination over the 12 assessment occasions (*p*s ≥ 0.289).

**Table 6:** Parameter estimates for the cohort model for rumination by gender.

		Women					Men				
Birth cohort		Est.	*SE*	*p*	95% CI		Est.	*SE*	*p*	95% CI	
					LB	UB				LB	UB
**1995–1991**	*i*	**3.27** ^***^	0.58	<0.001	2.13	4.41	**0.51**	0.95	0.594	−1.35	2.36
	*s*	**1.89** ^***^	0.51	<0.001	0.90	2.88	**−0.48**	0.82	0.556	−2.09	1.13
	*q*	**0.46** ^***^	0.11	<0.001	0.25	0.67	**−0.06**	0.18	0.719	−0.41	0.28
1990–1986	*i*	2.14^***^	0.29	<0.001	1.57	2.70	2.20^***^	0.43	<0.001	1.36	3.04
	*s*	1.12^***^	0.31	<0.001	0.50	1.73	1.24^**^	0.47	0.008	0.32	2.15
	*q*	0.32^***^	0.08	<0.001	0.16	0.48	0.35^**^	0.12	0.005	0.10	0.59
1985–1981	*i*	1.38^***^	0.15	<0.001	1.10	1.67	0.90^***^	0.24	<0.001	0.43	1.36
	*s*	0.58^**^	0.22	0.008	0.15	1.02	−0.08	0.36	0.819	−0.79	0.63
	*q*	0.22^**^	0.08	0.006	0.06	0.38	0.02	0.14	0.893	−0.25	0.28
1980–1976	*i*	1.05^***^	0.05	<0.001	0.95	1.15	1.08^***^	0.08	<0.001	0.93	1.24
	*s*	0.30^*^	0.13	0.016	0.06	0.55	0.32	0.19	0.101	−0.06	0.69
	*q*	0.18^*^	0.07	0.012	0.04	0.32	0.21	0.11	0.055	−0.01	0.43
1975–1971	*i*	0.89^***^	0.01	<0.001	0.86	0.92	0.89^***^	0.02	<0.001	0.85	0.93
	*s*	0.13^*^	0.05	0.010	0.03	0.23	−0.05	0.07	0.478	−0.20	0.09
	*q*	0.17^**^	0.06	0.009	0.04	0.29	0.00	0.09	0.976	−0.19	0.18
1970–1966	*i*	0.80^***^	0.01	<0.001	0.77	0.82	0.82^***^	0.02	<0.001	0.79	0.86
	*s*	−0.02	0.03	0.364	−0.08	0.03	−0.12^**^	0.04	0.001	−0.19	−0.05
	*q*	0.17^**^	0.06	0.003	0.06	0.29	0.22^**^	0.08	0.005	0.07	0.38
1965–1961	*i*	0.76^***^	0.03	<0.001	0.71	0.81	0.81^***^	0.03	<0.001	0.75	0.88
	*s*	−0.03	0.07	0.682	−0.17	0.11	−0.05	0.09	0.609	−0.23	0.14
	*q*	0.03	0.05	0.636	−0.08	0.13	−0.01	0.07	0.942	−0.14	0.13
**1960–1956**	*i*	**0.80** ^***^	0.07	<0.001	0.66	0.93	**1.01** ^***^	0.09	<0.001	0.83	1.18
	*s*	**−0.13**	0.12	0.289	−0.37	0.11	**−0.39** ^**^	0.16	0.012	−0.69	−0.09
	*q*	**0.03**	0.05	0.541	−0.07	0.14	**0.12**	0.07	0.060	−0.01	0.25
1955–1951	*i*	1.04^***^	0.16	<0.001	0.73	1.35	1.34^***^	0.19	<0.001	0.97	1.71
	*s*	−0.45^*^	0.20	0.021	−0.84	−0.07	−0.72^**^	0.23	0.002	−1.17	−0.26
	*q*	0.12^*^	0.06	0.044	0.00	0.24	0.18^*^	0.07	0.011	0.04	0.31
1950–1946	*i*	2.22^***^	0.36	<0.001	1.52	2.92	1.00^*^	0.41	0.015	0.20	1.80
	*s*	−1.56^***^	0.35	<0.001	−2.25	−0.88	−0.39	0.40	0.333	−1.17	0.40
	*q*	0.37^***^	0.08	<0.001	0.20	0.53	0.09	0.10	0.366	−0.10	0.27
1945–1941	*i*	−0.04	0.71	0.950	−1.44	1.35	0.53	0.79	0.505	−1.02	2.08
	*s*	0.49	0.56	0.389	−0.62	1.59	0.01	0.62	0.982	−1.21	1.24
	*q*	−0.09	0.11	0.406	−0.31	0.12	0.00	0.12	0.998	−0.24	0.24
1940–1936	*i*	0.70	1.45	0.627	−2.14	3.54	1.87	1.45	0.196	−0.97	4.71
	*s*	−0.22	0.96	0.821	−2.10	1.67	−0.96	0.96	0.315	−2.84	0.92
	*q*	0.05	0.16	0.737	−0.26	0.36	0.17	0.16	0.278	−0.14	0.48

Significant gender differences within birth cohorts denoted in bold. i, intercept; s, linear slope; q, quadratic slope; CI, confidence interval; LB, lower bound; UB, upper bound. Variances were constrained to equality across birth cohorts and genders (*i* = 0.51^***^, *SE* = 0.01; *s* = 0.02^***^, *SE* = 0.00; *q* = 0.00, *SE* = 0.00). ^***^p < 0.001, ^**^p < 0.01, ^*^p < 0.05.

Regarding men, only 4 of the 12 birth cohorts showed significant changes in rumination over time (see Table 6). As shown in Figure 2, these cohorts mirrored the findings for women in that the 1990–1986 cohort displayed curvilinear increases in rumination over time (*s* = 1.24, *SE* = 0.47, *p* = 0.008; *q* = 0.35, *SE* = 0.12, *p* = 0.005). The 1970–1966 cohort showed linear decreases that slowed over time (*s* = −0.12, *SE* = 0.04, *p* = 0.001; *q* = 0.22, *SE* = 0.08, *p* = 0.005), whereas the 1960–1956 cohort showed linear decreases (*s* = −0.39, *SE* = 0.16, *p* = 0.012) but no significant curvilinear change (*q* = 0.12, *SE* = 0.07, *p* = 0.060). Finally, the 1955–1951 birth cohort showed linear decreases in rumination, but this rate of change slowed over time (*s* = −0.08, *SE* = 0.011, *p* < 0.001; *q* = 0.01, *SE* = 0.004, *p* = 0.015).

To formally examine differences between women and men in the cohort model, we constrained the cohort estimates for women and men to equality within each birth cohort. Constraining all 11 birth cohorts to equality revealed that (some of) the birth cohorts differed in their trajectories over time by gender [*Wald*_(36)_ = 71.13, *p* < 0.001]. Subsequent comparisons of each birth cohort revealed differences between women and men among the 1995–1991 [*Wald*_(3)_ = 15.50, *p* = 0.001] and 1960–1956 [*Wald*_(3)_ = 9.58, *p* = 0.023] birth cohorts. The remaining birth cohorts did not demonstrate significant gender differences over time [*Wald*s_(3)_ = 0.87–6.72, *p*s ≥ 0.081], suggesting only small cohort-specific gender differences. Specifically, women in the 1995–1991 were generally higher in rumination (relative to men) and *increased* over time, while men in this cohort experienced (nonsignificant) *declines* in rumination. Similarly, in the 1960–1956 cohort, men reported significant declines in rumination, while women in this cohort remained relatively stable in rumination across time.

### 3.4. Model comparisons

While the aging, period, and cohort models all fit these data comparably (see Table 3), a visual inspection of the aging and cohort estimates in Figures 1, 2 reveals apparent aging effects for rumination among women and men, albeit with some very minor cohort differences. Namely, while the cohort estimates for women largely overlapped between birth cohorts (i.e., the 95% error bars for each point estimate largely overlapped; see Figure 1), there was slightly less overlap among the young birth cohorts. Moreover, the youngest cohort (1995–1991) demonstrated higher levels of rumination than estimated in the aging line and slightly less overlap with its adjacent birth cohort. Thus, these findings suggest a primarily normative developmental trajectory of rumination across the lifespan for women, with some limited evidence of cohort-based differences among young birth cohorts.

Concerning men, Figure 2 reveals a significant overlap between birth cohorts *and* between the broader cohort and aging models. That is, the cohort estimates largely follow the aging trajectory. As such, while there may be a distinct trajectory for the youngest birth cohort among women, these findings suggest that the course of rumination across the adult lifespan primarily reflects an aging process among men, irrespective of one’s birth cohort.

## 4. Discussion

The present study examined how rumination develops and changes across the adult lifespan using a large longitudinal nationwide random probability sample of adults over 12 years. Specifically, we utilized cohort-sequential latent growth modeling, which allowed us to inspect whether rumination follows a normative aging process across the lifespan or whether the development of rumination across time differed between cohorts. Critically, we examined trajectories of rumination over time among women and men separately, given that the extant literature suggests gender differences in rumination across different life stages (Nolen-Hoeksema, 1994; Nolen-Hoeksema and Aldao, 2011; Johnson and Whisman, 2013). In examining these processes, we aimed to elucidate gender differences in rumination (or lack thereof) across the lifespan, extending prior research that has predominantly focused on understanding the emergence rather than the continuity of gender differences in rumination.

Overall, our findings revealed that mean levels of rumination generally decreased from age 18 to 81, albeit at a rate that slowed over time. This trajectory appeared to reflect a normative aging process, corroborating previous research suggesting ruminative strategies are highest in young adulthood and decrease as one ages and gains life experience over time (e.g., Carstensen, 1987; Charles and Carstensen, 2007; Sütterlin et al., 2012; Zimmermann and Iwanski, 2014). Our findings, however, advance this understanding by demonstrating this normative trajectory in a large, nationwide sample of adults over 12 years. Indeed, previous research has predominantly focused on identifying the age groups most at “risk” for rumination rather than how rumination develops and changes across the lifespan. Our data provide a unique opportunity to explore this by directly comparing aging and cohort trajectories of rumination across time.

Interestingly, trajectories of rumination across the lifespan were relatively consistent across women and men, suggesting a common developmental trend irrespective of gender. That said, small gender differences did emerge in our aging model, and our period model revealed higher initial mean levels of rumination in the two youngest cohorts among women (relative to their male counterparts). Moreover, formal tests of gender differences in our cohort model revealed significant differences between women and men in the 1995–1991 birth cohort (and, to some extent, the 1960–1956 cohort). Thus, our findings align with previous research suggesting that young women face unique stressors that may predispose vulnerability to ruminative responding styles (e.g., Nolen-Hoeksema et al., 1999; Michl et al., 2013), and align with wider societal shifts in attitudes toward mental health over time (e.g., Pescosolido et al., 2021).

These findings suggest that gender differences in rumination may be better understood as age- and cohort-specific rather than as a consistent difference across the lifespan. While young female cohorts reporting the highest levels of rumination is perhaps unsurprising, these findings alleviate concerns that women are higher in rumination across the *entire* lifespan and that these patterns of responding may, in turn, put women at increased risk for depression and other mental health disorders (e.g., see Nolen-Hoeksema and Harrell, 2002; Johnson and Whisman, 2013; Rickerby et al., 2022). Although the differences between women and men in depressive disorders is well-established, these findings suggest that rumination may not play a significant role in this gender difference at later lifestages. Instead, the focus should be toward educating young women on the adverse consequences of rumination, and the benefits of active problem-solving strategies, to lessen the prevalence of adverse mental health in these groups.

Additionally, while both women and men experienced normative changes in rumination across the lifespan, more cohort differences emerged among women, suggesting that women’s reported rumination may be more sensitive to societal shifts than men’s and further supporting the assertion that women experience gender-specific events that impact their mental health (Nolen-Hoeksema et al., 1999). Given the generational shifts in how masculinity is defined and expressed, it is somewhat surprising, however, that young male cohorts were not considerably higher in rumination than their older counterparts (Anderson, 2018). Indeed, conformity to traditional masculine norms is associated with lower *reporting* of emotional distress (Ridge et al., 2011) and less willingness for men to seek mental health support (e.g., Pederson and Vogel, 2007; Lorber and Garcia, 2010; Johnson et al., 2012). However, changing priorities toward emotional intelligence and “soft skills” in contemporary society means that how men “perform” masculinity is changing (Gough, 2018). As such, one might expect young male cohorts to be more likely to disclose their emotional responses to stressors than their older counterparts. While our cohort model did not reveal any substantial cohort-based differences among men, these generational differences may only become apparent as young generations age and enter older life stages. Future research should consider this possibility and continue to examine how new generations of men navigate their emotions and mental health.

### 4.1. Limitations and future research directions

The present study contributes rare insights into the development of rumination across the lifespan. There are, however, limitations worthy of consideration. Namely, space constraints associated with a longitudinal survey restricted our assessment of rumination to a single item. Our measure of rumination was designed to assess general negative repetitive thoughts as directly as possible (see Nolen-Hoeksema et al., 1993), allowing us to examine general levels of rumination in the population. While we could not assess particular forms of rumination, our analyses provide insight into the development of general ruminative thinking across the lifespan. Nonetheless, it is possible that gender differences only emerge consistently for particular *forms* of rumination (e.g., brooding, reflecting, deliberate, and intrusive rumination) across the lifespan (see García et al., 2017) or in response to specific life domains (e.g., body image or interpersonal domains; see Mezulis et al., 2002). Future research should examine these possibilities using indicators of rumination with multiple items across different domains.

Although a strength of our study is the use of a large, nationwide random sample, our participants generally scored low on rumination across time which may contribute to the lack of gender differences. Mean rumination scores were well below the scale’s mid-point across the lifespan—as expected of a nonclinical population—with only 5.6–7.8% of the sample scoring above the mid-point at each wave. Thus, the relatively low levels of rumination in the sample may occlude gender differences, or these differences may only be apparent in clinical samples. Future research should consider this possibility when assessing the development and change in rumination across time, particularly when assessing gender or age differences.

Relatedly, although our study aimed to be representative of the New Zealand population, there were some discrepancies compared to the general population. Namely, our sample overrepresented women and New Zealand Europeans (see Sibley, 2023, for full information about the NZAVS sample). Concerning gender, our sample size for male participants is sufficient to detect change in rumination over time, and differences in sample sizes for women and men should not significantly affect our results. That said, the sample sizes for *older* male cohorts are small relative to other groups, which may occlude potential gender differences between women and men at older ages. Relatedly, recent research among transgender and gender-diverse individuals suggests a nuanced relationship between rumination and minority stress (e.g., Sarno et al., 2020) that we could not capture in our analyses. Future research should thus consider how rumination may develop and change differently across different identities and contexts, with particular attention to male and gender-diverse cohorts.

Additionally, our sample consisted of predominantly New Zealand Europeans, which may bias our findings in favor of Western WEIRD populations. Indeed, research suggests that the prevalence and consequences of rumination differ across cultures (e.g., Bonanno et al., 2005; Chang et al., 2010). These differences are primarily attributed to differences in maladaptive versus adaptive rumination (Grossmann and Kross, 2010), perceptions of change (Choi and Miyamoto, 2023), and holistic versus analytic thinking (De Vaus et al., 2018). Notably, research examining rumination among Māori (i.e., New Zealand’s Indigenous people) reveals that Māori with a greater sense of cultural efficacy report lower levels of rumination (Matika et al., 2017). Thus, culture and ethnicity are important factors that shape rumination. While examining cultural and ethnic-based differences in rumination is beyond the scope of the present study, we encourage future research to consider how cultural differences may impact developmental and cohort-based changes in rumination across the lifespan.

It is also important to note that we cannot fully distinguish between aging and cohort effects, as doing so would require extensive data across the lifespan *within* individuals. For example, cohort differences may be more (or, counterintuitively, less) pronounced if each cohort was followed across their entire lifespan. Likewise, although we provide some theoretical explanations for our findings, our approach does not elucidate *why* rumination declines as people age, nor can it explain the differences between birth cohorts. For example, rumination may decrease across the lifespan (or differ between birth cohorts) because of differences in life experiences and stressors (Kessler and McLeod, 1984; Kendler et al., 2001; Howard et al., 2022) or shifting attitudes toward mental health (e.g., Mojtabai, 2007; Rhydderch et al., 2016; Angermeyer et al., 2017). That said, such data is beyond the scope of this particular study. Nonetheless, our use of 12 annual assessments and a broad range of birth cohorts provides tentative evidence of the normative trajectory of rumination across the adult lifespan. These findings lay the critical foundations for future studies examining the mechanisms underlying the development and change of rumination as people age.

### 4.2. Conclusion

While the extant literature suggests gender differences in rumination persist across the lifespan, no research to date has directly examined this hypothesis. Likewise, research has yet to elucidate whether changes in rumination over time reflect a normative aging process or birth cohort differences. As such, the present study utilized multigroup cohort-sequential latent growth models to directly compare aging, period, and cohort effects in rumination across the adult lifespan (ages 18–81) and whether these trajectories differed between women and men. Our findings suggest a normative aging process whereby rumination is highest in young adulthood and declines across the adult lifespan, albeit at a rate that slows over time. This trajectory was largely similar among women and men. However, key cohort differences emerged among young women that suggest some context- and age-specific gender differences in rumination over time. Taken together, our results provide critical insights into the development of rumination in adulthood and demonstrate the importance of disentangling aging and cohort effects across the lifespan.

## Data availability statement

The data analyzed in this study is subject to the following licenses/restrictions: The dataset presented in this article is not readily available because ethical restrictions and the need to protect the confidentiality of study participants prevent public deposition of raw data. The data described in the paper are part of the New Zealand Attitudes and Values Study (NZAVS). Full copies of the NZAVS data files are held by all members of the NZAVS management team and advisory board. A de-identified dataset containing the variables analyzed in this manuscript is available upon request from the corresponding author or any member of the NZAVS advisory board for the purposes of replication or checking of any published study using NZAVS data. The Mplus syntax used to test all models reported in this manuscript are available on the NZAVS OSF page: https://doi.org/10.17605/OSF.IO/75SNB. Requests to access these datasets should be directed to CS, c.sibley@auckland.ac.nz.

## Ethics statement

The studies involving humans were approved by the University of Auckland Human Participants Ethics Committee (Reference Number: 22576). The studies were conducted in accordance with the local legislation and institutional requirements. The participants provided their written informed consent to participate in this study.

## Author contributions

KL wrote the original manuscript and performed the statistical analysis. CS organized the database and funding for the study. CH, EZ, and CS provided extensive feedback on the manuscript. All authors approved the manuscript in its final form.
